# French nationwide monthly online consultation meeting on complex abdominal wall repairs: 4-year assessment of an innovative collaborative decision-making tool

**DOI:** 10.1007/s10029-025-03483-9

**Published:** 2025-10-09

**Authors:** Gaëtan-Romain Joliat, Jean-François Gillion, Grégory Baud, Benoît Romain, Yohann Renard, Pablo Ortega-Deballon, Vincent Dubuisson, Haitham Khalil, David Moszkowicz, Guillaume Passot

**Affiliations:** 1 Department of Visceral Surgery, Lausanne University Hospital CHUV, University of Lausanne (UNIL), Lausanne, Switzerland; 2 Department of Visceral and Digestive Surgery, Antony Private Hospital, Antony, France; 3 Department of General and Endocrine Surgery, Lille University Hospital, Lille, France; 4 Department of General and Digestive Surgery, Hautepierre Hospital, Strasbourg, France; 5 Department of General, Digestive and Endocrine Surgery, Claude Cabrol University Hospital, Reims, France; 6 Department of Digestive Surgery, University Hospital of Dijon, Dijon, France; 7 Department of Digestive and Endocrine Surgery, Bordeaux University Hospital, Bordeaux, France; 8 Department of Digestive and Oncologic Surgery, Charles Nicolle University Hospital, Rouen, France; 9 Department of General and Digestive Surgery, Hôpital Louis Mourier, DMU ESPRIT - GHU AP-HP Nord - Université Paris Cité, Colombes, France; 10 Department of General Surgery and Surgical Oncology, Centre Hospitalier Universitaire Lyon Sud, 165, chemin du Grand Revoyet, Pierre-Bénite, 69495 France

**Keywords:** Hernia, Treatment, Optimization, Surgical techniques

## Abstract

**Purpose:**

Treatment of complex cases of abdominal wall reconstruction (AWR) has become more frequent, and management options are diverse. Treatment decision could be improved using a multicentric consultation meeting. The aim of this study was to assess quantitatively and qualitatively the outcomes of the French national online consultation meeting (OCM).

**Methods:**

The OCM was implemented in January 2021. This is an OCM where all surgeons working in France can present their cases of complex AWR for opinion. Descriptive statistics on this OCM were collected from implementation to March 2025. Moreover, a survey on how participating surgeons perceived this OCM was performed.

**Results:**

During the study period, a total of 436 cases were presented at the OCM (384 ventral hernias, 88% and 52 groin hernias, 12%). Overall, 127 surgeons participated in the OCM. The majority of presented patients originated from university hospitals (*n* = 294, 68%).

Thirty-one surgeons (31/127 = 24%) who participated in the OCM answered the survey. Most surgeons found that the OCM had a very high pedagogical interest (median 9/10, IQR 8–10). In total, 30/31 surgeons (97%) would recommend to a colleague to take part in this OCM. Among surgeons who presented a case and answered the survey (*n* = 24), 1 (4%), 10 (42%), and 13 (54%) surgeons found the OCM useful, very useful, and indispensable, respectively. All surgeons who responded were satisfied with the OCM (21 were very satisfied, 87%).

**Conclusions:**

Implementation of a nationwide OCM for complex AWR is feasible and sustainable. Feedbacks from participants emphasized the usefulness of this meeting designed to help surgeons to better tailor treatment to patients with complex hernias. However, as patient outcomes were not available, precluding any analyses on the impact of the OCM on patient postoperative evolution, further follow-up results will be needed.

**Supplementary Information:**

The online version contains supplementary material available at 10.1007/s10029-025-03483-9.

## Introduction

Patients with complex ventral hernias are nowadays more often encountered [1]. This multifaceted complexity results from the development and validation of optimization tools such as preoperative progressive pneumoperitoneum, botulinum toxin injection, or multimodal prehabilitation, and new surgical techniques such as anterior or posterior component separation, parastomal hernia repairs, or minimally invasive (laparoscopic or robotic) procedures [1–7]. These recent advances have enlarged treatment opportunities and options, enabling to repair some complex abdominal wall cases, previously inaccessible to surgical repair. However, not all abdominal hernias are treatable and not all hernias require treatment. Multidisciplinary meetings might help to determine which hernias are reasonable to treat with the best outcomes for the patients, and how to optimize pre- and postoperative care and perioperative planification. Recent publications have underlined the effectiveness of multidisciplinary meetings on the pre- and postoperative management of complex abdominal wall repairs (CAWR) [8–10].

Additionally, the choice of the appropriate surgical technique is pivotal in CAWR. The experience of current various operative techniques and tools may differ from one surgical team to another. All above-mentioned arguments emphasize the need of a multicenter overview or discussion in the shared decision-making of complex cases.

Online meetings allow for such remote discussions and collaboration between surgeons from different hospitals. Four years ago, a nationwide online consultation meeting (OCM) on complex ventral and groin hernia cases was implemented in France.

The aim of the present study was to evaluate the 4-year performance of such an innovative nationwide monthly OCM and to assess its role in the decision-making for complex ventral or groin hernia cases.

## Methods

### Study design

This study consisted in a 4-year qualitative and quantitative assessment of a nationwide monthly OCM on ventral and groin hernia complex cases, launched in January 2021.

## Inclusions

All cases of abdominal wall reconstruction for ventral or groin hernias deemed as complex by the presenting surgeons were accepted for discussion. The complexity of the case was subjectively judged by the presenting surgeon and did not always comply with the published definition of a complex hernia [11]. All registered cases from January 2021 to March 2025 were included in the present study.

## Variables of interest

The quantitative analysis was based on the key metrics of the OCM (number of presenting and participating surgeons, number of cases, hospital types, and geographical locations). The qualitative analysis was based on the results of a satisfaction survey among the participating and presenting surgeons. The survey is detailed in the **Supplementary Material**.

## Consultation meeting principles

This nationwide monthly OCM is directed by the French academic abdominal wall and hernia surgery society (SFCP-CH) and the Club-Hernie registry. This OCM was developed in compliance with the French administrative principles (https://www.has-sante.fr/jcms/c_2806878/fr/reunion-de-concertation-pluridisciplinaire) for such consultation meetings.

This OCM is open to any identified French-speaking surgeons who may need advice on the medical and surgical care of one of their CAWR patients.

Surgeons formalized their request on the SFCP-CH website (https://sfcp-ch.fr). After verification of the surgeon’s hospital affiliation, the latter is provided with credentials to log in to the Club-Hernie registry where he/she registers the deidentified patient data, a clinical summary (history, physical examination, and imaging results) and clearly indicates the main question(s) to discuss. The anonymized clinical imaging of the patient is screen-shared during the meeting. Surgeons must be present during the presentation of their patient. All cases to present are added in the meeting patient list, and a Zoom© link is provided to all potential participants.

A 120-minute online meeting is organized monthly. At the end of the peer discussion of each case a recommendation summarizing the suggestions is written in real time by one of the meeting organizers, which can then be downloaded in PDF format by the presenting surgeon. This conclusion is not a compelling injunction but rather a peer-given piece of advice.

After the meeting the presenting surgeon is asked to complete the Club Hernie registry with the intra- and postoperative data (if patient has been operated on) and to indicate whether he has followed the OCM advice or not and why.

## Ethics

This study is only based on de-identified registered data. The registry is compliant with the GDPR and the French CNIL (N° 2212908).

### Statistics

Continuous variables were presented as medians with interquartile ranges (IQR), and discrete variables were presented as numbers with percentages. All statistical analyses were performed using SPSS 29.0 for Mac OS (IBM, Chicago, USA).

## Results

### Key metrics of the consultation meeting

From January 2021 to March 2025, 436 cases (384 ventral hernias, 88% and 52 groin hernias, 12%) were presented at the OCM by a total of 127 participating surgeons.

**Fig. 1:**
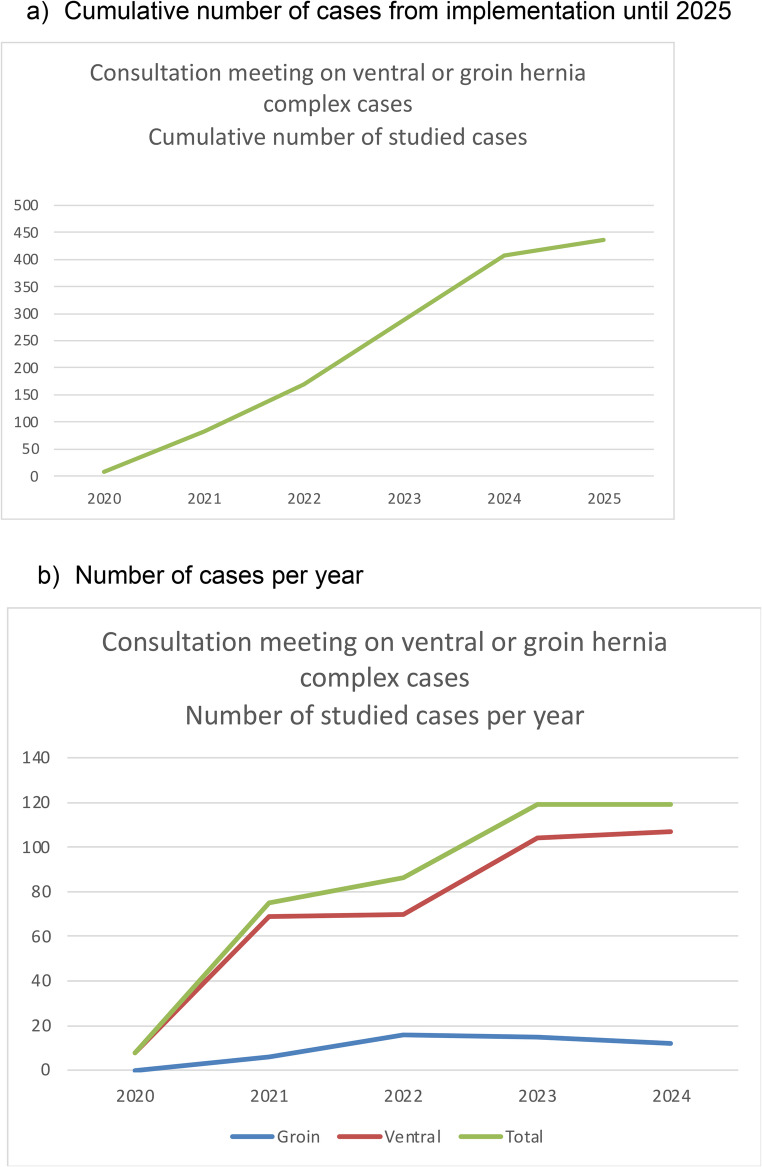
Cumulative number of cases from implementation until 2025

The cumulative number of cases has quickly grown to currently reach 436 cases (Fig. 1a). The annual number of cases increased every year until 2023, and then asymptotically stabilized around 120 cases/year which corresponds to nearly 12 cases studied per monthly meeting (Fig. 1b). As the meeting duration was approximately 120 min, the mean time spent for each case was about 10 min. The annual number of studied complex ventral hernia repairs has been growing while the annual number of registered complex groin hernia repairs remained low and relatively stable (Fig. 1b).

**Fig. 2:**
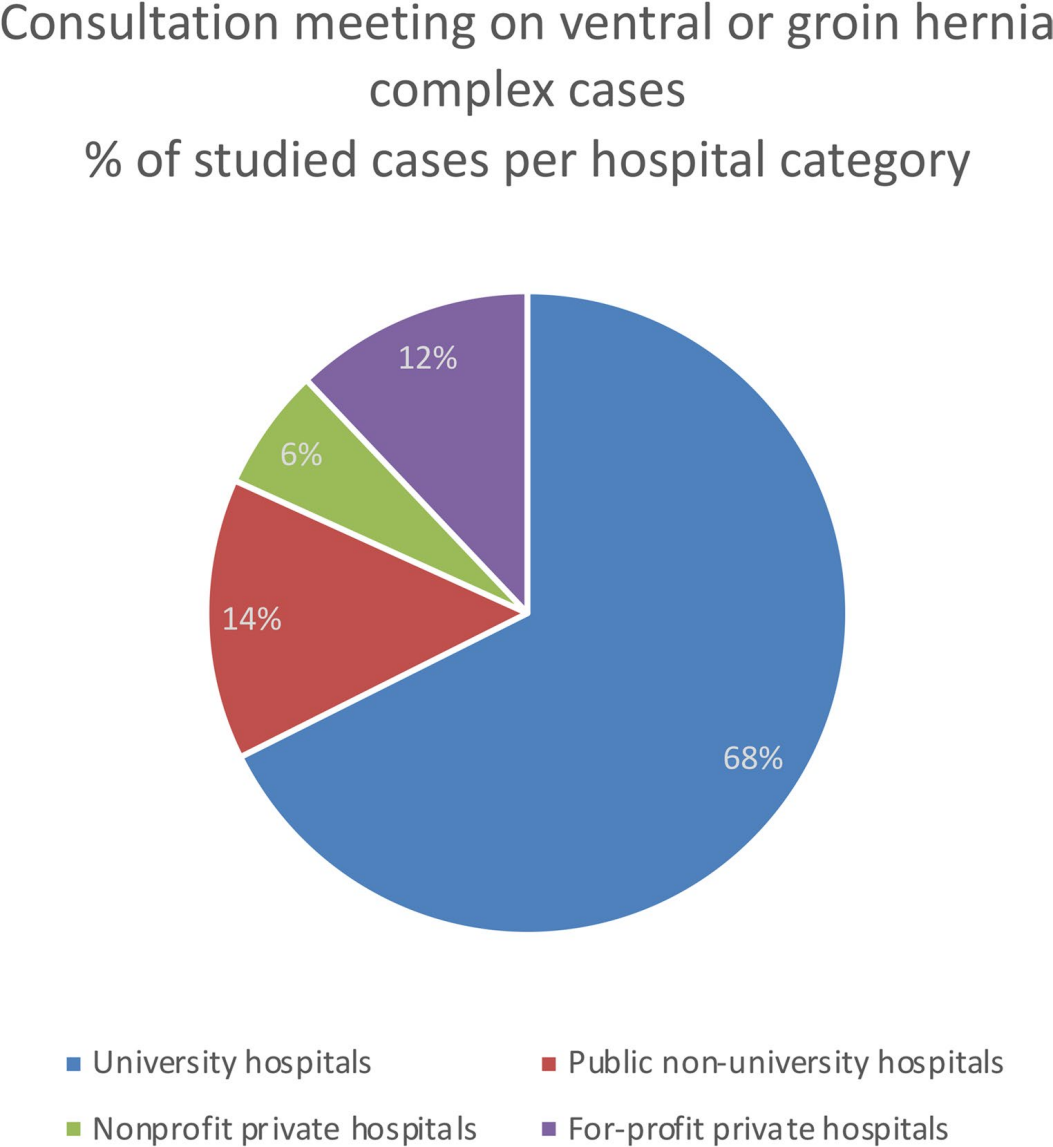
Percentage of cases according to the hospital categories

**Fig. 3:**
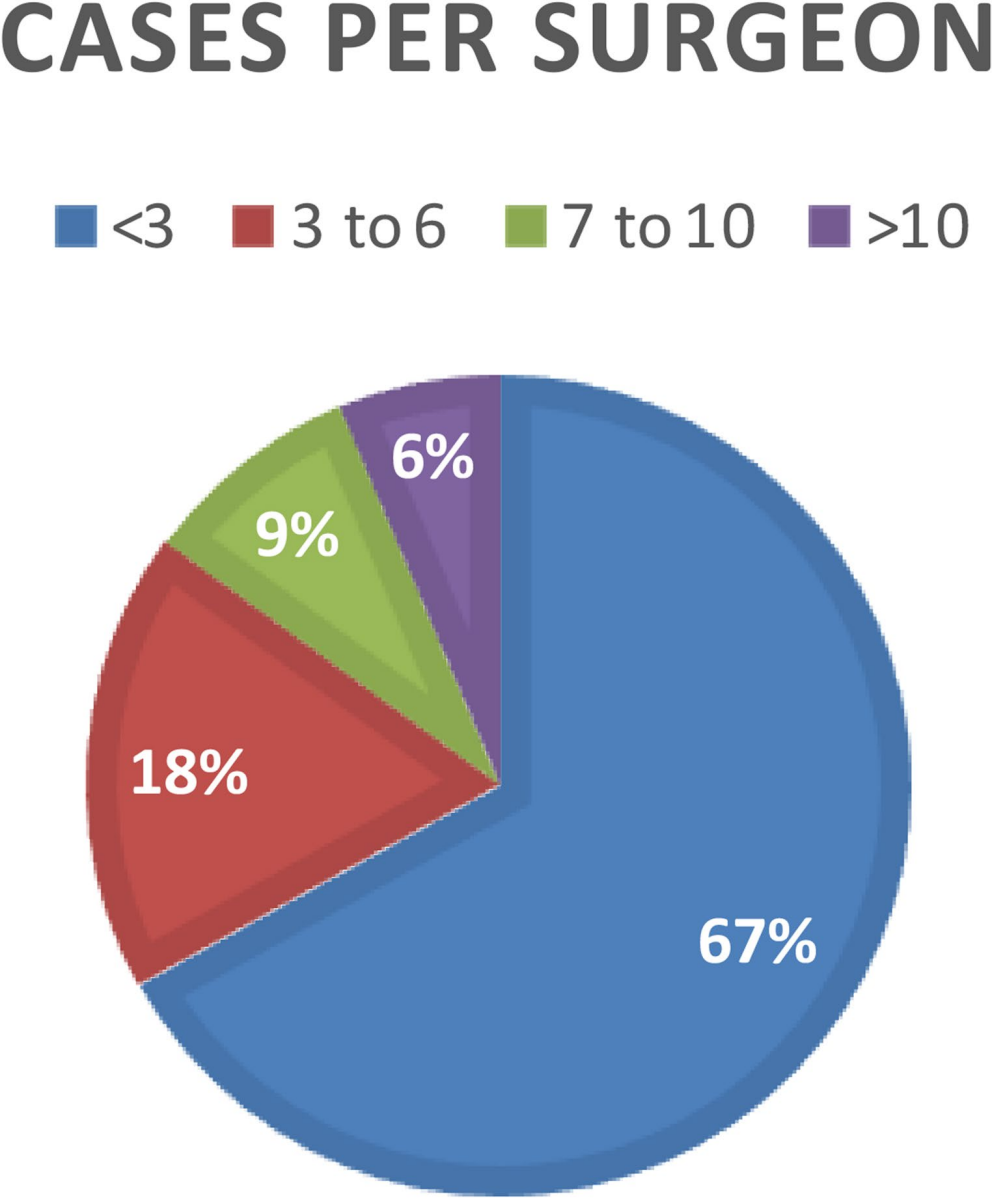
Number of presented cases per surgeon during the study period

The large majority of the studied cases were from public hospitals (*n* = 356, 82%), mainly university hospitals (*n* = 294, 68%, Fig. 2). Almost one fourth of the complex cases of groin hernia repairs were presented by surgeons working in private hospitals (12/52 = 23%), whereas only 41/384 ventral hernia cases (11%) originated from private clinics. Most of the 127 participating surgeons (67%) presented less than 3 cases each (Fig. 3). Only 6% of surgeons presented more than 10 complex cases each during the study period (Fig. 3).

**Fig. 4:**
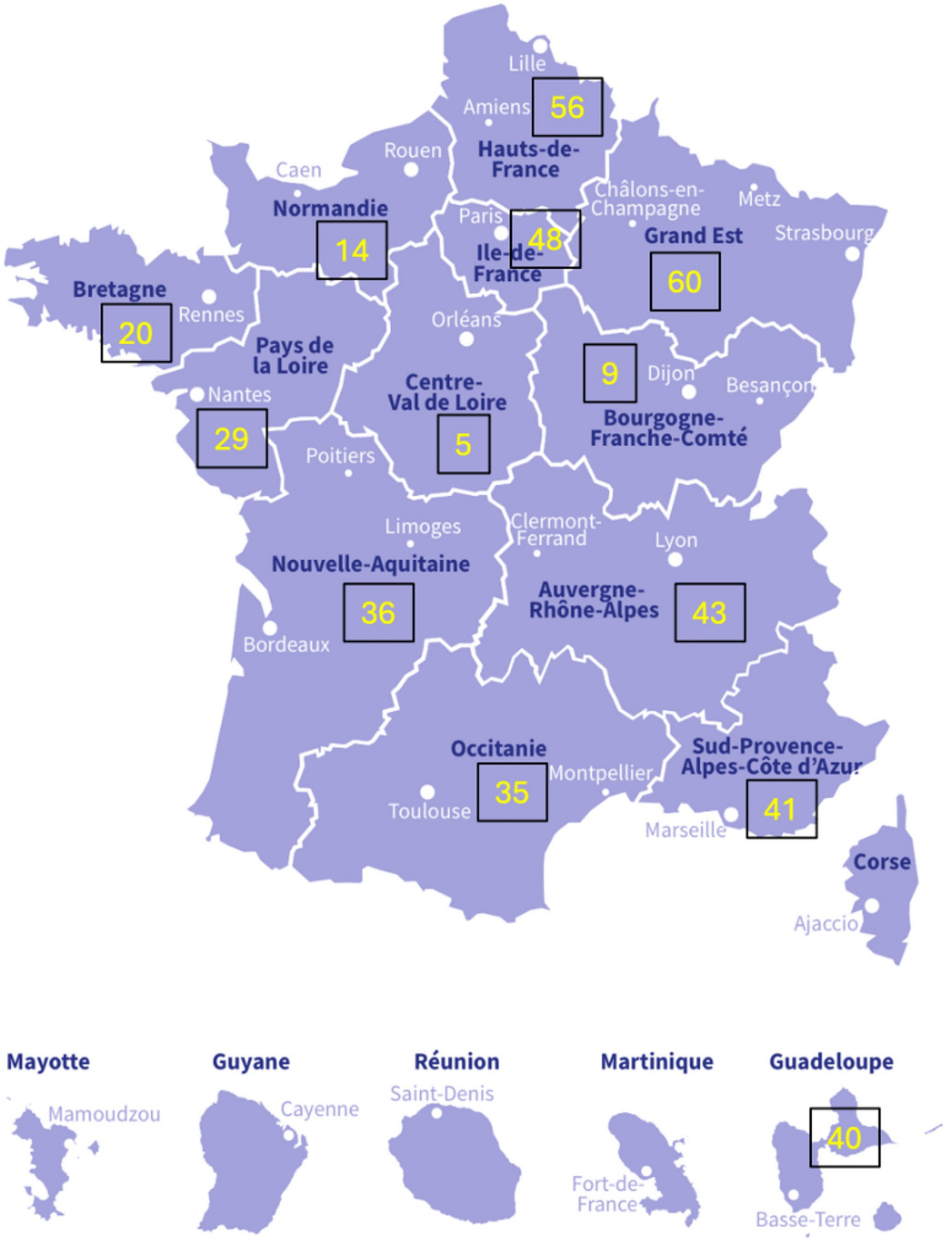
Geographical distribution of the presented cases (French regions)

Presented cases were evenly distributed and originated from all regions of France, even from Guadeloupe (Fig. 4). The meeting schedule (Paris-zone 6 to 8 pm) was compatible with an active participation of our overseas colleagues (40 cases presented).

Except for Guadeloupe, highly involved in this meeting, the ratio of presented cases divided by the region population ranged from 0.15 to 1 per 100,000 inhabitants over the 4 years of the study.

### Qualitative analysis

This OCM fulfilled the French administrative principles for such meetings. Based on a formal organization, with a dedicated secretary, a coordinating team maintaining patient and participating surgeon lists and scheduled meetings, 11 monthly meetings per year were organized since the launch of the meeting 4 years ago. All scheduled meetings took place on time, none was postponed.

All invited surgeons from all parts of France could connect, share imaging sequences and participate in the meeting. The meeting summaries written in real time during the meeting, contained a clear therapeutic proposal or an alternative, were edited in PDF form, being inserted on demand in the patient records.

Thirty-one surgeons (31/127 = 24%) who participated to the OCM (presenters and non presenters) answered the survey completely or in parts. Among the surgeons who responded to the survey (*n* = 31), 11 worked in a private hospital/clinic, 10 in a non university hospital, and 5 in a university hospital. The rest were still in training. Most surgeons found that the consultation meeting had a very high pedagogical interest (median 9/10 on a 0 to 10 interest scale, IQR 8–10). In total, 30/31 surgeons (97%) responded yes when asked if they would recommend to a colleague to take part in this meeting. Survey results regarding quality of clinical presentation, quality of technical discussion, conviviality, and pedagogical interest are summarized in Table 1.

**Table 1:** Results of the survey answered by surgeons who assisted at least to one consultation meeting with or without presentation of a patient (n=31)

Item	Median or number	Interquartile range or %
Quality of the clinical presentations	9	8–10
Quality of the technical discussions	9	8–10
Conviviality of the discussions	10	9–10
Pedagogical value of the meeting	9	8–10
Do you think that this online meeting permits to have the opinion of several specialists at once? Yes No	24 7	77% 23%
Do you think that this online meeting is better than an individual discussion with a specialist? Yes No	30 1	97% 3%

Responses to the survey part evaluating the relevance, format, and satisfaction of the meeting by surgeons who presented at least one patient (*n* = 24) are depicted in Table 2. The data highlighted the perceived value of the multidisciplinary discussion in influencing clinical decision-making. Notably, all respondents reported that the meeting at least sometimes raised unexpected points, and 88% acknowledged that it suggested at least sometimes an unexpected surgical technique. Furthermore, for 96% of cases, the recommended technique was performed (often or always), and in 96% of cases, intraoperative findings were often or always concordant with the meeting’s expectations. Importantly, the vast majority of surgeons (96%) found the meeting to be either very useful or indispensable, and 87% reported being very satisfied with the experience.

**Table 2:** Results of the survey answered by surgeons who presented at least one patient to the consultation meeting (n=24)

Item	Median or number	Interquartile range or %
Number of patients presented 1 to 4 5 to 9 10 or more	16 7 1	67% 29% 4%
Did the meeting raise unexpected points? Sometimes Often Always	12 10 2	50% 42% 8%
Did the meeting suggest an unexpected management? Never Sometimes Often	2 12 10	8% 50% 42%
Did the meeting suggest an unexpected surgical technique? Never Sometimes Often Always	3 10 10 1	12% 42% 42% 4%
Did the meeting confirm your initial therapeutic choice? Sometimes Often Always	3 17 4	12% 71% 17%
Did the meeting summary include a clear therapeutic proposition? Sometimes Often Always	1 4 19	4% 17% 79%
Did you follow the recommendation of the meeting? Often Always	5 19	21% 79%
Did you have to refer the patient for the operation? Never Sometimes Often Always	12 10 1 1	50% 42% 4% 4%
Were the intraoperative findings concordant with the previsions of the meeting? Sometimes Often Always	1 13 10	4% 54% 42%
Did you perform the technique recommended during the meeting? Sometimes Often Always	1 11 12	4% 46% 50%
For your next case of complex hernia will you present it at the meeting? Probably Certainly	6 18	25% 75%
At a national level, do you think this meeting is Useful? Very useful? Indispensable?	1 10 13	4% 42% 54%
How satisfied are you with the meeting? Satisfied Very satisfied	3 21	13% 87%

## Discussion

This appraisal study on a nationwide OCM for CAWR 4 years after its implementation showed that the yearly number of presented cases has increased during the first 3 years after implementation and is now steady. Moreover, participants to the OCM expressed a high level of satisfaction, highlighting the success of this online reunion.

While multidisciplinary meetings have been organized for many years in oncology, and more recently in other surgical fields such as for example bariatric surgery, such meetings remain rare in abdominal wall surgery. A Dutch surgical team reported the effectiveness of multidisciplinary team meetings (MDT) in decision-making regarding prehabilitation and planned intensive care unit admission after CAWR [10]. A MDT decision for a planned ICU admission after CAWR (232 patients) was more accurate than any of the other risk-stratifying tools [10]. The same team reported that prehabilitation allowed patients with relevant comorbidities and risk factors to achieve the same postoperative results as patients without those risk factors [8]. The indication to undertake a preconditioning program might be effective at the discretion of an MDT. Other centers also described their experience with the implementation of MDT in CAWR, showing safety of such MDT recommendations and reporting the possibility to objectively evaluate risk factors, to facilitate decision-making, to provide guidance for patient care, and to improve education through these MDT [9, 12]. These MDT often are monocentric meetings but multidisciplinary. The current implemented national OCM is on the contrary monodisciplinary (only surgeons) but multicentric (surgeons from different hospitals) focusing on the risk-benefit balance of operating the presented patient, the selection of the best preparation method, and the choice of the best surgical technique. The discussion is not limited to one surgical team but encompasses many different specialized surgical teams with broad experience. As mentioned in other publications, such meetings permit to substantiate a collective decision and relieve surgeons from being isolated and having to take a decision on their own [8, 9].

As shown in Fig. 2, the presented cases mainly came from tertiary care academic hospitals (294 cases, 68%), while other cases came from general (62 cases, 14%), private (53 cases, 12%), non-profit private (27 cases, 6%) hospitals. This repartition underlines the distribution variety of such cases and the requirement to individualize, if needed, referral to CAWR centers geographically distributed over the territory to cover the population needs.

Management and treatment of ventral hernia have become more complex [1, 13]. Complexity lies in the multimorbidity of the patients, including risk factors for complications and recurrences, in the patient preoptimization, and in the technical aspects of the reconstruction [13–15]. In the present article, complexity was sometimes different than the published definitions because each individual presenting surgeon subjectively judged a case as complex based on his/her technical capacities, own experience, and available resources. This is an important point to highlight. OCM should indeed allow surgeons with different expertise to have the opportunity to present a case, even though it might be judged as non complex by an expert in CAWR.

The present study showed that a nationwide OCM had a high level of satisfaction among the participating surgeons (87% were very satisfied and all surgeons were satisfied or very satisfied). Moreover, 97% of surgeons would recommend this OCM to a colleague. These findings underline that the format of the OCM is appreciated by the participating surgeons. Additionally, the number of surgeons participating in this meeting (*n* = 127 over 4 years, presenters and non-presenters) underlines the pedagogical interest of the meeting. The pedagogical value of the OCM was indeed highlighted in the performed survey (median 9/10, IQR 8–10). This meeting is not only interesting and educative for residents or young surgeons, but also for more experienced surgeons who can benefit of continuous medical education via these real-life patient cases. This finding corroborates conclusions of other publications on MDT underpinning this important didactical notion [16, 17]. These findings support the utility of such meetings in refining surgical strategy and enhancing clinical confidence, particularly in complex hernia management.

To contextualize the French surgical landscape, 2309 general surgeons were registered in 2025 according to the statistical unit of the French Ministry of Health. No specific hernia centers exist in France. However, expert hernia units are part of general surgery departments. There is currently a dozen of hernia units with a couple of surgeons working in these units and performing CAWR.

79% of surgeons (19/24) said that they always followed the OCM recommendations, while 21% (5/24) said that they often followed the recommendations (Table 1). These results indicate that the OCM recommendations were almost always followed, which highlights that consensual opinions of experts were respected by the surgeons who presented their case at the meeting. Of note, among the 5 surgeons who answered often, 2 of them estimated that the intraoperative findings were always concordant with the OCM previsions and 3 of them estimated that the intraoperative findings were often concordant with the OCM previsions.

Regarding quality of the recommendations, only one surgeon reported (1/24 = 4%) that the intraoperative findings were sometimes (compared to 13/24: often and 10/24: always) in agreement with the previsions of the OCM. This result emphasizes the quality and relevance of the recommended propositions of the meeting.

This study has some limitations that need to be acknowledged. Regarding the quality part of the study, a relatively low number of surgeons participated in the survey, which can induce some selection and response bias. Several hypotheses can be postulated for this response rate. Surgeons who participated in the meeting only once were potentially less interested in filling in the survey. Additionally, most of the 127 surgeons who received the survey presented < 3 cases, which could play a role in the commitment to answering the survey. Other common explanations such as lack of time, responder demographics, misunderstanding of the survey purpose, or survey fatigue can be cited. Furthermore, data regarding postoperative outcomes of patients who were presented at the OCM were not available, precluding any analyses on the impact of the OCM on patient postoperative evolution. Additionally, not all surgeons completed if they followed the meeting advice and if not the reasons for that.

Future perspectives regarding the development of the OCM and research could be to obtain more precise data on the outcomes and follow-up of patients who were presented. It could also be interesting to perform another survey to specifically highlight what could be improved in the OCM process. Regarding the OCM development, it could be interesting to spread the meeting to other French-speaking regions or countries (e.g., Switzerland or Belgium) and enriching to invite other specialties such as radiologists or physiotherapists to participate.

In conclusion, this 4-year evaluation study showed that a nationwide OCM for CAWR is feasible and sustainable on a regular basis with considerable increase of the number of patients presented since implementation. Moreover, this reunion was found to be well appreciated and very useful by general and abdominal wall surgeons in France who answered the survey (24% response rate, risk of selection bias). 

## Supplementary Information

Below is the link to the electronic supplementary material.

Supplementary Material 1 (DOCX. 222 KB)
