# Supplementary material for: French nationwide monthly online consultation meeting on complex abdominal wall repairs: 4-year assessment of an innovative collaborative decision-making tool
Source: Hernia. 2025 Oct 9;29(1):288. doi: 10.1007/s10029-025-03483-9 (PMC12511159; doi:10.1007/s10029-025-03483-9)
Supplement: Supplementary file 1 — Supplementary Material 1 (DOCX. 222 KB) [file 10029_2025_3483_MOESM1_ESM.docx]

**Survey on the national online consultation meeting for complex abdominal wall hernia**

Are you still in training?

Yes

No

Where do you work?

University hospital

Community hospital

Private clinic

Quality of the clinical presentations (0-10)

Quality of the technical discussions (0-10)

Conviviality of the discussions (0-10)

Pedagogical value of the meeting (0-10)

Do you think that this online meeting permits to have the opinion of several specialists at once?

Yes

No

Do you think that this online meeting is better than an individual discussion with a specialist?

Yes

No

Number of patients presented

1 to 4

5 to 9

10 or more

Did the meeting raise unexpected points?

Never

Sometimes

Often

Always

Did the meeting suggest an unexpected management?

Never

Sometimes

Often

Always

Did the meeting suggest an unexpected surgical technique?

Never

Sometimes

Often

Always

Did the meeting confirm your initial therapeutic choice?

Never

Sometimes

Often

Always

Did the meeting summary include a clear therapeutic proposition?

Never

Sometimes

Often

Always

Did you follow the recommendation of the meeting?

Never

Sometimes

Often

Always

Did you have to refer the patient for the operation?

Never

Sometimes

Often

Always

Were the intraoperative findings concordant with the previsions of the meeting?

Never

Sometimes

Often

Always

Did you perform the technique recommended during the meeting?

Never

Sometimes

Often

Always

For your next case of complex hernia will you present it at the meeting?

Probably

Certainly

At a national level, do you think this meeting is

Not useful?

Useful?

Very useful?

Indispensable?

How satisfied are you with the meeting?

Unsatisfied

Satisfied

Very satisfied
